# Multiple pulmonary nodules with diffuse idiopathic pulmonary neuroendocrine cell hyperplasia and minute pulmonary meningothelial‐like nodules

**DOI:** 10.1002/rcr2.1344

**Published:** 2024-04-03

**Authors:** Tsuyoshi Sasada, Ryo Tachikawa, Shigeo Hara, Keisuke Tomii

**Affiliations:** ^1^ Department of Respiratory Medicine Kobe City Medical Center General Hospital Kobe Japan; ^2^ Department of Pathology Kobe City Medical Center General Hospital Kobe Japan

**Keywords:** carcinoid tumours, diffuse idiopathic pulmonary neuroendocrine cell hyperplasia, meningothelial‐like nodules, multiple pulmonary nodules, tumourlets

## Abstract

A 78‐year‐old woman presented with multiple pulmonary nodules, mixed with solid and ground‐glass nodules. We pathologically confirmed that the multiple pulmonary nodules were a combination of diffuse idiopathic pulmonary neuroendocrine cell hyperplasia (DIPNECH) and multiple pulmonary meningothelial‐like nodules (MPMNs). This is the first case report of concurrent DIPNECH and MPMNs.

## Graphical abstract

We report the first case report of concurrent diffuse idiopathic pulmonary neuroendocrine cell hyperplasia (DIPNECH) and multiple pulmonary meningothelial‐like nodules (MPMNs). A 78‐year‐old woman presented with multiple pulmonary nodules, mixed with solid and ground‐glass nodules. Pathology confirmed that the multiple pulmonary nodules were a combination of (DIPNECH) and (MPMNs).

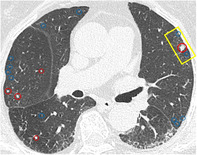

## CLINICAL IMAGE

**FIGURE 1:**
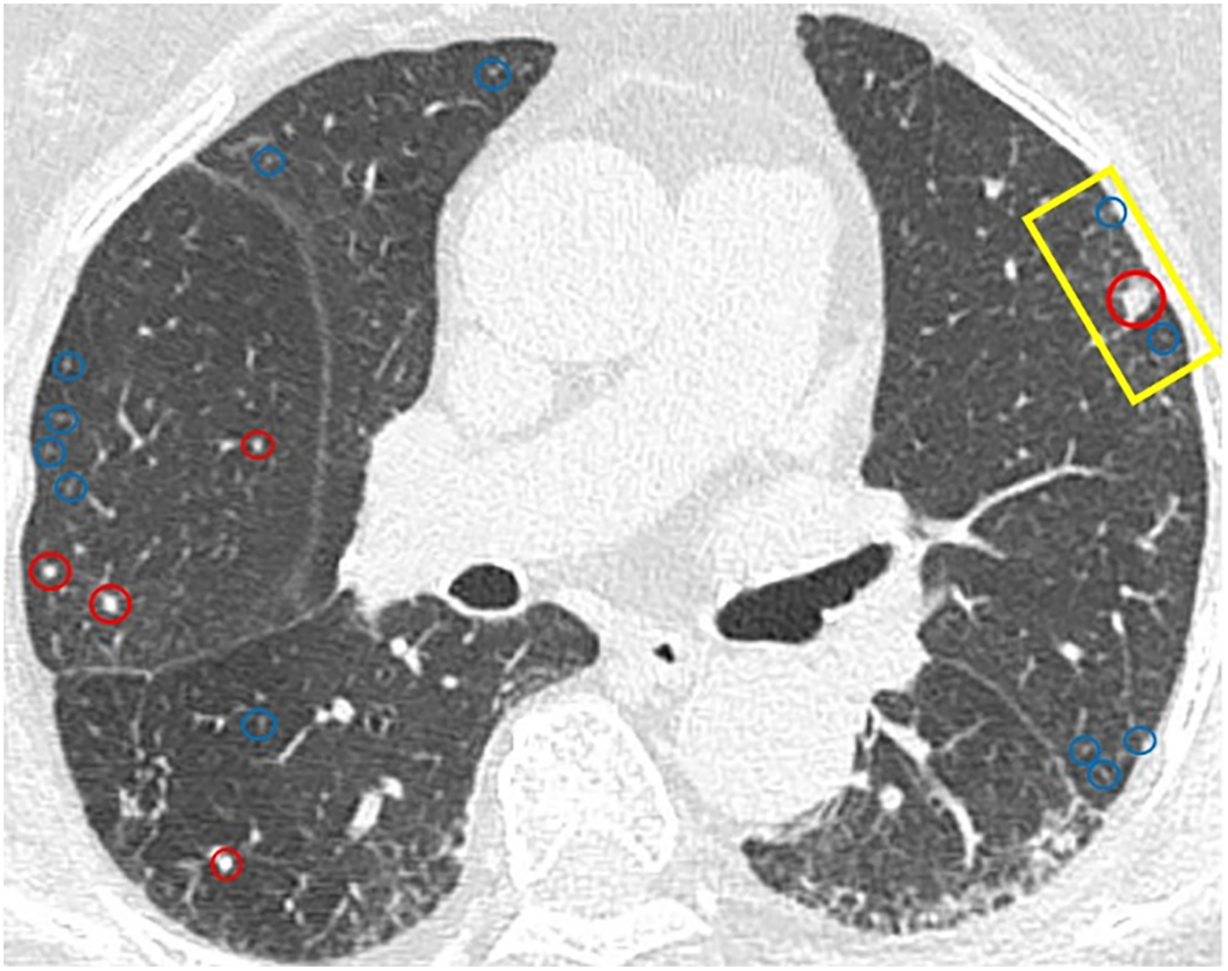
Axial slice of the chest computed tomography; areas marked with red circles represent solid nodules, and those marked with blue circles denote ground‐glass nodules. The territory marked by the yellow square is targeted for biopsy.

**FIGURE 2:**
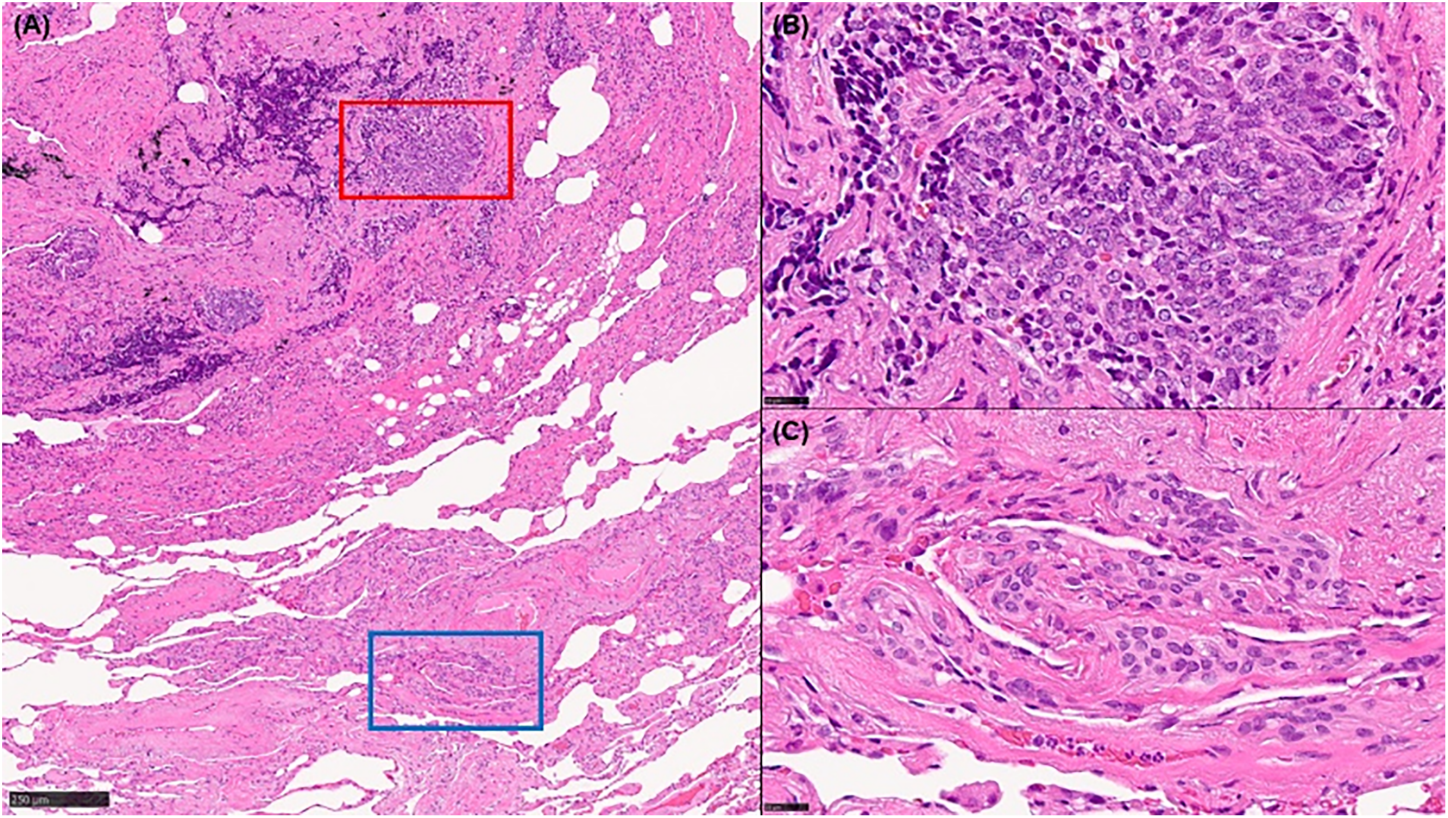
(A) Overview of the pathological specimen; the red square represents a tumourlet, and the blue square denotes a minute pulmonary meningothelial‐like nodule. (B) Tumourlets. (C) Minute pulmonary meningothelial‐like nodules. (Haematoxylin and eosin staining; A: ×4; B: ×40; C: ×40).

**FIGURE 3:**
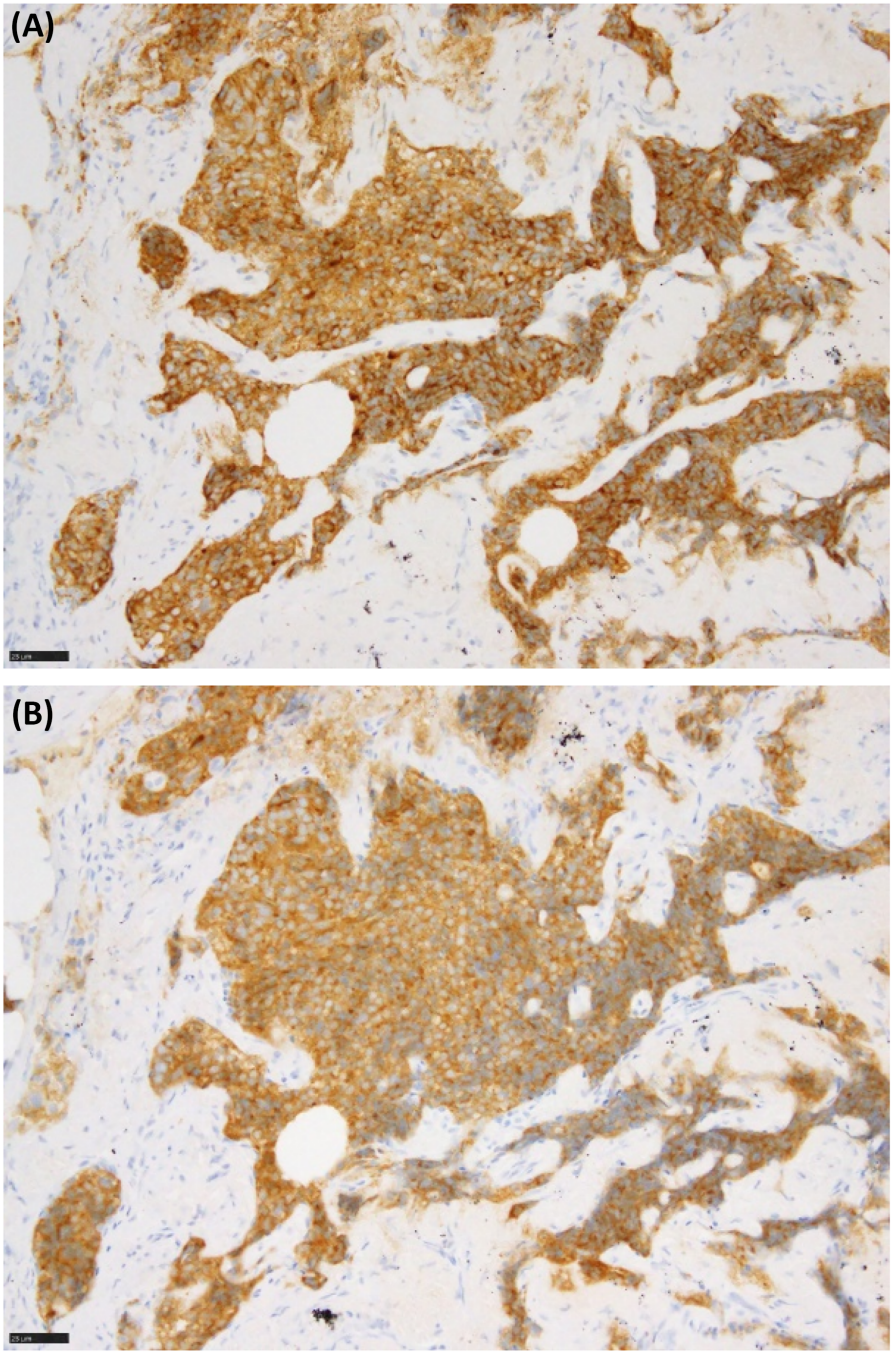
(A) Positive chromogranin A stain (×40 magnification). (B) Positive synaptophysin stain (×40 magnification).

We report a 78‐year‐old woman with persistent dyspnoea and cough, lacking smoking history, respiratory diseases, or exposure to inhaled antigens. High‐resolution computed tomography (CT) revealed multiple pulmonary nodules, approximately 5 mm, mixed with solid and ground‐glass (Figure 1). Pulmonary function tests were within normal limits. Video‐assisted thoracoscopic lung biopsy detected tumourlets and minute pulmonary meningothelial‐like nodules (MPMNs), suggesting mixed pathology in multiple pulmonary nodules (Figure 2A–C). These tumourlets were positive for chromogranin A and synaptophysin, which are specific for neuroendocrine cells (Figure 3A,B). CT findings of the solid nodules were compatible with tumourlets and carcinoid tumours, whereas ground‐glass nodules were consistent with MPMNs.^1^, ^2^ Blood tests revealed elevated progastrin‐releasing peptide levels (599 pg/mL). The CT image and specimen exhibited multiple tumourlets and carcinoid tumours, leading to a clinical diagnosis of diffuse idiopathic pulmonary neuroendocrine cell hyperplasia (DIPNECH). DIPNECH is considered a preinvasive lung lesion within the spectrum of neuroendocrine cell neoplasia.^1^ To our best knowledge, this is the first report of concurrent DIPNECH and MPMNs. MPMNs may relate to several non‐malignant diseases^2^; nonetheless, their connection to DIPNECH remains unclear. This case emphasizes the need to evaluate diverse pathologies in patients with pulmonary nodules and different CT findings.

## AUTHOR CONTRIBUTIONS

Tsuyoshi Sasada wrote the initial manuscript and prepared the CT images. Ryo Tachikawa supervised the study and reviewed the manuscript. Shigeo Hara prepared pathological images. Keisuke Tomii was responsible for conceptualization. All the authors contributed to the writing, review, and final approval of the manuscript.

## CONFLICT OF INTEREST STATEMENT

None declared.

## ETHICS STATEMENT

The authors declare that appropriate written informed consent was obtained for the publication of this manuscript and accompanying images.

## Data Availability

The data that support the findings of this study are available on request from the corresponding author. The data are not publicly available due to privacy or ethical restrictions.
